# AMFGNN: an adaptive multi-view fusion graph neural network model for drug prediction

**DOI:** 10.3389/fphar.2025.1543966

**Published:** 2025-04-28

**Authors:** Fang He, Lian Duan, Guodong Xing, Xiaojing Chang, Huixia Zhou, Mengnan Yu

**Affiliations:** ^1^ Faculty of Pediatrics, The Chinese PLA General Hospital, Beijing, China; ^2^ Department of Child Growth and Development Clinic, The Seventh Medical Center of PLA General Hospital, Beijing, China; ^3^ National Engineering Laboratory for Birth Defects Prevention and Control of Key Technology, Beijing, China; ^4^ Beijing Key Laboratory of Pediatric Organ Failure, Beijing, China; ^5^ Department of Pediatric Surgery, The Seventh Medical Center of PLA General Hospital, Beijing, China

**Keywords:** drug prediction, drug-disease association prediction, graph attention network, contrastive learning, Kolmogorov-Arnold network

## Abstract

**Introduction:**

Drug development is a complex and lengthy process, and drug-disease association prediction aims to significantly improve research efficiency and success rates by precisely identifying potential associations. However, existing methods for drug-disease association prediction still face limitations in feature representation, feature integration, and generalization capabilities.

**Methods:**

To address these challenges, we propose a novel model named AMFGNN (Adaptive Multi-View Fusion Graph Neural Network). This model leverages an adaptive graph neural network and a graph attention network to extract drug features and disease features, respectively. These features are then used as the initial representations of nodes in the drug-disease association network to enable efficient information fusion. Additionally, the model incorporates a contrastive learning mechanism, which enhances the similarity and differentiation between drugs and diseases through cross-view contrastive learning, thereby improving the accuracy of association prediction. Furthermore, a Kolmogorov-Arnold network is employed to perform weighted fusion of various final features, optimizing prediction performance.

**Results:**

AMFGNN demonstrates a significant advantage in predictive performance, achieving an average AUC value of 0.9453, which reflects the model‘s high accuracy in prediction.

**Discussion:**

Cross-validation results across multiple datasets indicate that AMFGNN outperforms seven advanced drug-disease association prediction methods. Additionally, case studies on Hepatoblastoma, asthma and Alzheimer‘s disease further confirm the model‘s effectiveness and potential value in real-world applications.

## 1 Introduction

Since the outbreak of the COVID-19 pandemic, the global healthcare system has faced unprecedented challenges (Vaz et al., 2023; Meng et al., 2024), making the need for safe and effective treatment strategies more urgent than ever. Drug repositioning has attracted widespread attention because of its ability to rapidly identify new therapeutic options, effectively reducing both the cost and timeline of new drug development (Pushpakom et al., 2019; Tang et al., 2023). Recent breakthroughs in high-throughput screening technologies and continuous improvements in computational methods have significantly enhanced the efficiency and accuracy of computer-aided drug repositioning in identifying potential drug-disease associations (Singh et al., 2024; Zeng et al., 2024).

In the field of drug-disease association (DDA) prediction, research methods have gradually evolved from traditional machine learning models to deep learning techniques (Lavecchia, 2015). Traditional machine learning-based DDA prediction identifies potential drug-disease associations through data modeling and feature extraction, and the application of diverse algorithms has further expanded the depth of research in this area. For example, Gao et al. (2022a) combined similarity fusion technology with Laplacian regularization algorithms to accurately predict new indications for drugs and diseases; Yang et al. (2021) developed the MKDGRLS model, employing a multi-kernel approach and Laplacian regularization to handle complex interactions and optimizing model parameters through alternating least squares; additionally, Zhang et al. (2020) proposed the Bayesian inductive matrix completion (DRIMC) method, which integrates features from multiple data sources for analysis in latent space, effectively predicting new drug applications; Niu et al. (2024) introduced the SRR-DDI model, which utilizes a self-attention mechanism to represent drug substructures finely and incorporates drug similarity features, significantly enhancing the stability and performance of drug interaction predictions.

Compared to traditional methods, drug-disease association (DDA) prediction methods based on graph neural networks (GNNs) have made significant breakthroughs in recent years (Zhang et al., 2021; Meng et al., 2024; Zeng et al., 2024). Their unique advantage lies in the ability of GNNs to effectively process multimodal data and model complex network structures, a characteristic that has also gained attention in other fields (Liu et al., 2023). For example, Gao et al. (2022b) proposed the CTST model, which constructs a heterogeneous network of drugs and diseases, using graph convolutional autoencoders to encode shared and unique features of nodes. The model also integrates features through an attention mechanism, significantly improving prediction accuracy. Zhao et al. (2022a) developed the HINGRL model, which leverages a heterogeneous information network encompassing drug-disease and protein-protein interactions, enhances feature recognition through graph representation learning, and combines a random forest algorithm for precise drug indication prediction. Yang et al. (2024) designed the GCNGAT model, which integrates graph convolutional networks with graph attention networks, particularly suitable for drug repositioning. This model analyzes drug-disease associations by constructing heterogeneous graphs and extracts key interaction features in multi-disease contexts. Additionally, Liu et al. (2024) proposed the AMDGT framework, which uses a dual-graph transformer technique to integrate similarity data and complex biochemical information, deeply merging drug and disease features to efficiently predict potential drug associations. Wang et al. (2025) proposed an automatic collaborative learning framework that integrates neighbor interaction metrics with the message-passing mechanism of Graph Neural Networks to enhance prediction accuracy.

Although machine learning and deep learning methods have made significant progress in drug-disease association (DDA) prediction, existing approaches still face challenges in handling complex, multidimensional data and efficiently integrating information from multiple perspectives. To address this issue, we propose an Adaptive Multi-view Fusion Graph Neural Network (AMFGNN) model. The model first constructs drug-drug similarity networks and disease-disease similarity networks, using graph attention networks to extract drug and disease features, which are then used as initial features for the downstream drug-disease association network nodes. To further improve the accuracy of drug-disease association prediction, the model incorporates a contrastive learning mechanism that enhances the similarity and dissimilarity between drugs and diseases through cross-view contrastive learning. The final features are predicted using a Kolmogorov-Arnold Networks (KAN), further improving the prediction accuracy of drug-disease associations. By integrating multi-source heterogeneous information and achieving adaptive weighted fusion, the model flexibly handles complex drug and disease data, dynamically adjusting the weights of different information sources, thereby improving the accuracy, stability, and generalization of predictions. This approach establishes deeper connections between multidimensional data and multi-view information, providing stronger support for drug discovery and personalized medicine.

## 2 Materials and methods

**FIGURE 1:**
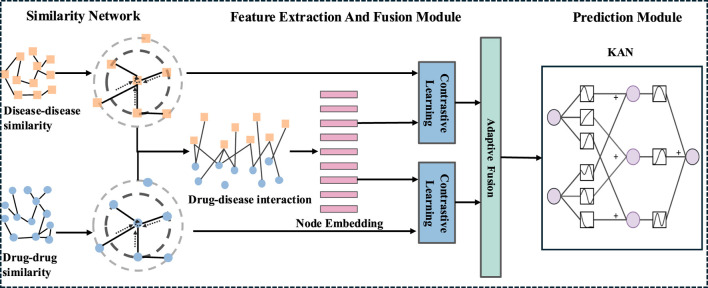
Illustration of the AMFGNN. The process begins with the construction of a similarity network, followed by feature extraction and fusion using the Graph Attention Network (GAT) and Adaptive Fusion Layer with contrastive learning. Finally, the prediction module employs Kolmogorov-Arnold Networks (KAN) for the final prediction.

In this section, we first describe the benchmark datasets used in the proposed model. Next, we introduce the AMFGNN model framework, which consists of three main components. As shown in Figure 1, the framework includes: (i) construct similarity network, (ii) feature extraction and fusion module, and (iii) prediction module.

### 2.1 Dataset

To comprehensively evaluate the performance of the proposed model, we adopted three benchmark datasets: Gottlieb et al. (2011), Luo et al. (2016), and Liang et al. (2017), which are widely used in drug repositioning research. Fdataset is a validated dataset containing 1,632 known drug-disease associations, involving 468 drugs and 298 diseases, providing a reliable reference standard for drug repositioning studies. Cdataset includes 663 drugs, 409 diseases, and 2,352 drug-disease interaction pairs, with the data first appearing in Luo et al.’s study. LRSSL consists of 3,051 validated drug-disease associations, involving 763 drugs and 681 diseases, and has been widely applied in drug repositioning research.

### 2.2 Graph attention networks

In this study, Graph Attention Network (GAT) (Veličković et al., 2017) is introduced for drug-disease prediction tasks. Graph Neural Networks (GNNs) (Wu et al., 2020) learn interactions between nodes and edges in a graph to perform tasks such as node classification, graph classification, and link prediction. GAT extends this by assigning different weights to each node and its neighbors using an attention mechanism. These weighted features are aggregated to learn the node’s embedding representation. We use a three-layer twin GAT network to extract features for both drugs and diseases. Taking the drug similarity network as an example, the drug similarity graph consists of a feature matrix $F_{a}$ and an adjacency matrix $A_{a}$ . To construct the adjacency matrix $A_{a}$ , we use a k-nearest neighbors (KNN) algorithm to identify the K most similar drugs to a drug $a_{i}$ , and establish connections. For drugs that do not belong to the K nearest neighbors, no connection is made.

In GAT, the attention mechanism computes the importance of a drug $a_{j}$ for its neighboring drug $a_{i}$ .Specifically, the attention coefficient between drug $a_{i}$ and its neighbor $a_{j}$ is calculated as follows Equation 1:

$$\alpha =\frac{exp\left(\text{LeakyReLU}\left(Wh_{i}\|Wh_{j}\right)\right)}{\sum_{k\in N_{e}\left(a_{i}\right)}exp\left(\text{LeakyReLU}\left(Wh_{i}\|Wh_{k}\right)\right)}$$ (1)

Here, W is the learnable weight matrix, and $h_{i}$ and $h_{j}$ represent the feature vectors of drugs $a_{i}$ and $a_{j}$ , respectively. The LeakyReLU function serves as the activation function, and the attention coefficient $\alpha_{ij}$ represents the importance of neighboring drug node $a_{j}$ to the central drug node $a_{i}$ . A higher value of $\alpha_{ij}$ indicates a greater contribution from the features of node $a_{j}$ to updating the representation of node $a_{i}$ . Through this mechanism, GAT effectively identifies and emphasizes connections in the drug similarity network that carry higher predictive significance, thus enhancing the quality of drug embedding representations and ultimately improving the accuracy of drug-disease association predictions.

After computing the attention coefficients, the feature vector of drug $a_{i}$ is updated by aggregating the features of all its neighboring drugs weighted by the attention coefficients. The updated feature vector $\hat{h_{i}}$ is calculated as follows Equation 2:

$$\hat{h_{i}}=\sigma \left(\underset{j\in Ne\left(a_{i}\right)}{\sum }\alpha Wh_{j}\right)$$ (2)

where σ is the activation function, and LeakyReLU is used. To capture the complex relationships between nodes, GAT introduces a multi-head attention mechanism that learns multiple sets of attention weights. The multi-head attention calculation is expressed as Equation 3:

$$\hat{h_{i}}={\|}_{k=1}^{K}\sigma \left(\underset{j\in N_{e}\left(a_{i}\right)}{\sum }\alpha_{ij}^{k}W^{k}h_{j}\right)$$ (3)

Here, K is the number of attention heads, and $\alpha_{ij}^{k}$ represents the weight of the k -th attention head between drug $a_{i}$ and drug $a_{j}.W^{k}$ is the weight matrix for the k -th attention head.

The multi-head attention mechanism allows GAT to capture diverse relationships between drugs from multiple perspectives, alleviating information bottlenecks and enhancing the model’s generalization ability. To integrate information from multiple heads, GAT averages the outputs of the different heads in the final layer, providing a more comprehensive and stable embedding representation Equation 4:

$$\hat{h_{i}}=\frac{1}{K}\overset{K}{\underset{k=1}{\sum }}{\hat{h_{i}}}^{k}$$ (4)

Through this multi-head attention mechanism, GAT can more effectively capture the complex dependencies between drugs and generate accurate drug embeddings. In this study, both the drug similarity and disease similarity networks employ a three-layer GAT structure to improve the accuracy of drug-disease prediction and the model’s expressive power.

### 2.3 Adaptive fusion

The core idea of the Graph Attention Network (GAT) is to update node features based on the importance weights of neighboring nodes. However, as the depth of the GAT network increases, an issue of over-smoothing may arise, where the features of all nodes in the graph become too similar, severely affecting the model’s prediction accuracy. To address this issue, we introduce residual connections to ensure that GAT can adaptively retain the original features while updating node features. The specific operation is as follows Equations 5‐8:

$$R\left(H_{as}^{0}\right)=\text{Elu}\left(W_{a}H_{as}^{0}+b_{a}\right)$$ (5)

$$R\left(H_{ds}^{0}\right)=\text{Elu}\left(W_{d}H_{ds}^{0}+b_{d}\right)$$ (6)

$$R_{ad}=\left(R\left(H_{as}^{0}\right);R\left(H_{ds}^{0}\right)\right)$$ (7)

$$R\left(H_{ad}^{0}\right)=\text{Elu}\left(W_{ad}H_{ad}^{0}+b_{ad}\right)=\text{Elu}\left(W_{ad}\left(H_{as}^{0};H_{ds}^{0}\right)+b_{ad}\right)=\left(R_{a};R_{d}\right)$$ (8)

Here, R (⋅) denotes the residual connection operation, $H_{as}^{0}$ denotes the initial feature representation of the drug similarity network (i.e., the pre-processed embedding derived from the original drug feature matrix $F_{a}$ ), while $H_{ds}^{0}$ represents the initial feature representation of the disease similarity network (i.e., the pre-processed embedding derived from the original disease feature matrix $F_{d}$ ). By introducing residual connections, the model adaptively preserves initial feature information, effectively addressing the issue of over-smoothing that typically occurs as the depth of the network increases. $H_{ad}^{0}$ corresponds to the feature of the drug-disease association view. The weight matrix is represented by $W_{k}$ , where $k\in \left\{a,d,ad\right\},b_{k}$ is the bias term, and $R_{k}$ denotes the residual result. The activation function used is the Elu function.

We fuse the residual connections of different views with the features from the previous layer, as shown in the following Equations 9‐11:

$$H_{a}^{\left(1\right)}=\epsilon_{1}H_{as}^{l}+\left(1-\epsilon_{1}\right)R_{a}$$ (9)

$$H_{d}^{\left(1\right)}=\epsilon_{2}H_{ds}^{l}+\left(1-\epsilon_{2}\right)R_{d}$$ (10)

$$H_{ad}=\epsilon_{3}H_{ad}^{l}+\left(1-\epsilon_{3}\right)R_{ad}=\left(H_{a}^{\left(2\right)};H_{d}^{\left(2\right)}\right)$$ (11)

Here, $H_{a}^{\left(q\right)}$ denotes the feature vector of the q -th view of the drug, $H_{as}^{l}$ is the feature representation of the drug similarity modality in the final layer of GAT, and $\epsilon_{i}$ is an adaptive variable learned during training, used to control the fusion weight between residual and original features.

To effectively integrate multi-view features of drugs and diseases, we adopt an adaptive feature fusion strategy Equations 12, 13:

$$H_{a}=\left[\eta_{1}H_{a}^{\left(1\right)}+\left(1-\eta_{1}\right)H_{a}^{\left(2\right)};H_{a}^{\left(1\right)};H_{a}^{\left(2\right)}\right]$$ (12)

$$H_{d}=\left[\eta_{2}H_{d}^{\left(1\right)}+\left(1-\eta_{2}\right)H_{d}^{\left(2\right)};H_{d}^{\left(1\right)};H_{d}^{\left(2\right)}\right]$$ (13)

Here, $H_{a}$ is the drug embedding after multi-view fusion, $H_{d}$ is the disease embedding after multi-modal fusion, and $\eta_{i}$ is the adaptive variable learned during model training. Finally, we input the fused drug embedding $M_{i}$ and disease embedding $D_{j}$ into KAN to compute the predicted potential connection ${\hat{S}}_{ij}$ Equation 14.

$${\hat{S}}_{ij}=M_{i}^{T}\cdot D_{j}$$ (14)

During training, we use the cross-entropy loss function to minimize the error between the model’s predictions and the true labels Equation 15:

$$L_{CE}=-\underset{\left(i,j\right)\in x^{+}\cup x^{-}}{\sum }\left[S_{ij}\ln{\hat{S}}_{ij}+\left(1-S_{ij}\right)\ln\left(1-{\hat{S}}_{ij}\right)\right]$$ (15)

Here, $x^{+}$ and $x^{-}$ represent the positive and negative sample sets in the dataset, respectively, and $S_{ij}$ is the true association score between drug $a_{i}$ and disease $d_{j}$ . To further improve the model’s prediction accuracy and constrain the parameter updates across modalities, we introduce multi-view contrastive learning as a regularization term. This method reduces the distance between features of the same sample from different views, while increasing the distance between features of different samples from different views, thereby enhancing the model’s feature representation ability. The objective function is defined as follows Equation 16:

$$dis\left(x,x^{+}\right)\ll d\left(x,x^{-}\right)$$ (16)

For each drug sample $a_{i}$ , the contrastive learning loss function is defined as Equation 17:

$$L_{MC}=\frac{1}{N}\overset{N}{\underset{j=1}{\sum }}\left(dis\left(m_{i}^{1},m_{i}^{2}\right)-dis\left(m_{i}^{1},m_{j}^{2}\right)\right)$$ (17)

Here, N is the total number of drug samples, $m_{i}^{p}$ represents the embedding of drug sample $a_{i}$ in the p -th modality, and d (u, v) is the distance function calculated using cosine similarity Equation 18:

$$dis\left(u,v\right)=-\frac{u^{T}v}{\left\|u\|\cdot \|v\right\|}$$ (18)

Similarly, for each disease sample $d_{j}$ , the loss function is defined as Equation 19:

$$L_{DC}=\frac{1}{N}\overset{N}{\underset{k=1}{\sum }}\left(dis\left(d_{j}^{1},d_{j}^{2}\right)-dis\left(d_{j}^{1},d_{k}^{2}\right)\right)$$ (19)

Finally, the overall loss function of the model is defined as Equation 20:

$$L=L_{CE}+\omega_{MC}L_{MC}+\omega_{DC}L_{DC}$$ (20)

### 2.4 Kolmogorov-Arnold Networks

In the drug repositioning task, to improve the parameter efficiency of the model, we modified the traditional multilayer perceptron (MLP) structure by replacing the final MLP module with Kolmogorov-Arnold Networks (KAN). KAN introduces learnable activation functions, replacing traditional linear weight matrices, which significantly enhance the network’s expressive power while maintaining or even improving model performance. The traditional MLP captures complex mappings through linear transformations and fixed nonlinear activation functions, which often limits model flexibility and increases parameter redundancy. Mathematically, an MLP is expressed as Equation 21:

$$MLP\left(Z\right)=\left(W_{K-1}◦\sigma ◦W_{K-2}◦\sigma \ldots ◦W_{1}◦\sigma ◦W_{0}\right)Z$$ (21)

Where Z is the input vector, $W_{k}$ is the weight matrix, and σ is the activation function. Although effective in learning complex functions, the fixed linear transformations in MLPs may restrict the adaptability of the network. KAN, in contrast, utilizes learnable nonlinear activation functions instead of fixed linear weight matrices, providing greater flexibility in capturing complex relationships between input features. Specifically, each connection in KAN is modeled by a combination of a parametric basis function and B-spline functions Equation 22:

$$f\left(x\right)=f\left(x_{1},\ldots ,x_{n}\right)=\overset{2n+1}{\underset{q=1}{\sum }}\Phi_{q}\left(\overset{n}{\underset{p=1}{\sum }}\phi_{q,p}\left(x_{p}\right)\right)$$ (22)

Each layer’s $\Phi_{i}$ consists of a set of learnable activation functions, represented as Equation 23:

$$\Phi =\left\{\phi_{q,p}\right\},\;p=1,2,\ldots ,n_{in},\;q=1,2,\ldots ,n_{\mathrm{out}}$$ (23)

We assume that a KAN can be expressed as $\left[n_{0},n_{1},\ldots ,n_{L}\right]\;n_{i}$ represents the number of neurons in the i -th layer. We use (l,i) to denote the i -th neuron in the l -th layer and $X_{\left(l,i\right)}$ to represent the activation value of the neuron (l,i) . Between the l -th and (l+1) -th layers, there are $n_{l}\times n_{l+1}$ activation functions: the activation function connecting (l,i) and (l+1,i) is represented as Equation 24:

$$\phi_{l,j,i},\;l=0,1,\ldots ,L-1,\;i=1,2,\ldots ,n_{l},\;j=1,2,\ldots ,n_{l+1}$$ (24)

The pre-activation value of $\phi_{l,j,i}$ is $x_{\left(l,i\right)}$ , and the post-activation value of $\phi_{l,j,i}$ is represented as Equation 25:

$${\hat{x}}_{l,j,i}=\phi_{l,j,i}\left(x_{l,i}\right)$$ (25)

The activation value of neuron $x_{\left(l+1,i\right)}$ is the sum of all incoming post-activation values Equation 26:

$$x_{l+1,j}=\overset{n_{l}}{\underset{i=1}{\sum }}{\hat{x}}_{l,j,i}=\overset{n_{l}}{\underset{i=1}{\sum }}\phi_{l,j,i}\left(x_{l,i}\right),\;j=1,\ldots ,n_{l+1}$$ (26)

The activation function ϕ is composed of a weighted sum of a basis function b(x) and a B-spline function spline(x) Equations 27‐29:

$$\phi \left(x\right)=w_{b}b\left(x\right)+w_{s}\text{spline}\left(x\right)$$ (27)

$$b\left(x\right)=\text{SiLU}\left(x\right)=\frac{x}{1+e^{-x}}$$ (28)

$$\text{spline}\left(x\right)=\underset{i}{\sum }c_{i}B_{i}\left(x\right)$$ (29)

Here, $w_{b}$ and $w_{s}$ represent the weights of the basis function and B-spline function, respectively. $c_{i}$ are the trainable parameters in the B-spline function, and $B_{i}\left(x\right)$ is the B-spline basis function defined on a grid. By using adaptive activation functions, KAN significantly enhances the representational power of neural networks, allowing the model to learn smoother and more complex transformations without increasing model complexity excessively. This flexible representation is particularly beneficial for integrating diverse features from multi-view data, such as drug similarity, disease similarity, and their interactions, leading to improved predictive performance for drug-disease associations.

## 3 Results and discussion

### 3.1 Parameter settings

We perform 10-fold cross-validation to evaluate the performance of AMFGNN. In the 10-fold cross-validation, all known and unknown drug-disease associations are randomly divided into 10 subsets of approximately equal size. Each subset is used as the test set in turn, while the remaining nine subsets serve as the training set.

We set the feature embedding size to 128 to achieve the best prediction performance in drug-drug similarity, disease-disease similarity, and drug-disease association graphs, and we set the dropout rate to 0.2 to optimize the training process of the network layers. For the graph model selection, we choose to use a 3-layer GAT instead of GCN (Graph Convolution Network) because GAT outperforms GCN in terms of AUC. The 3-layer structure helps prevent both information redundancy and over-smoothing issues. For optimization, we employed the Adam optimizer with a learning rate of 0.001 and weight decay of 0.001, training the model for 300 epochs.

### 3.2 Model evaluation and cross-validation

To rigorously evaluate the performance of our proposed model, we employed a standard 10-fold cross-validation approach. Specifically, drug-disease associations from each benchmark dataset were randomly divided into ten subsets of approximately equal size. In each fold, nine subsets were combined to form the training set, while the remaining subset served as the test set for evaluating the model’s performance. This procedure was repeated ten times, with each subset serving as the test set exactly once. We reported the model’s performance using the average and standard deviation across these ten evaluations. Furthermore, this cross-validation procedure was independently conducted on all three benchmark datasets (Fdataset, Cdataset, and LRSSL) to ensure the reliability and generalizability of our results.

### 3.3 Baseline methods

To evaluate the performance of AMFGNN, we performed 10-fold cross-validation on three public datasets: Fdataset, Cdataset, and LRSSL. The models compared in this study include LBMFF, SCPMFDD, SCPMF, MKGCN, and MNGACDA.

• LBMFF is a model for drug-disease relationship prediction that combines latent bilinear matrix factorization and focal loss. The model captures latent associations between drugs and diseases through matrix factorization techniques and introduces focal loss to address the class imbalance problem, enhancing the model’s ability to handle sparse data and hard-to-predict instances (Kang et al., 2023).
• SCPMFDD is a semi-supervised learning model for drug-disease prediction. The model combines collaborative projection matrix factorization and semi-supervised learning strategies to enhance prediction performance by leveraging known drug-disease relationships and unlabeled data (Li et al., 2022).
• SCPMF is a semi-supervised learning model for drug-disease prediction. It learns the latent relationships between drugs and diseases through collaborative matrix factorization, while also utilizing semi-supervised learning to enhance the model’s learning capability by incorporating unlabeled data. (Meng et al., 2021).
• MKGCN is a model for complex drug-disease prediction tasks. By introducing multiple kernel functions (Multi-Kernel), it integrates different types of graph structure features. MKGCN uses Graph Convolutional Networks (GCN) to process drug-disease graph data and applies kernel functions to weight different features, thereby more accurately capturing the complex relationships between drugs and diseases and improving the model’s ability to model and predict (Cui et al., 2023).
• MNGACDA is a graph neural network model for drug-disease prediction. The model combines multi-node graph attention mechanisms and dual attention mechanisms, effectively processing the drug and disease relationship graph through Graph Convolutional Networks (Yang and Chen, 2023).
• DDAGDL (Zhao et al., 2022b) is a model for drug–disease prediction that applies geometric deep learning over heterogeneous information networks. It integrates biological information into the network structure and uses an attention mechanism to learn effective representations of drugs and diseases, enabling improved performance on non-Euclidean biomedical data.
• RGLDR (Zhao et al., 2025) combines regulation-aware graph representation learning with meta-path-based connectivity patterns to capture diverse regulatory mechanisms in heterogeneous biological networks. It enhances drug and disease embeddings using a multi-view attention mechanism and predicts drug-disease associations with an XGBoost classifier. Experimental results demonstrate its superior performance over state-of-the-art methods on benchmark datasets.

According to the results shown in Table 1, AMFGNN achieves the highest AUC in all three datasets. AMFGNN demonstrates outstanding performance across different datasets. On the F dataset, the AUC value of AMFGNN is 0.9328, significantly higher than other models such as LBMFF (0.7953), SCPMFDD (0.7740), and others. Similarly, on the C dataset and LRSSL dataset, AMFGNN also shows higher prediction accuracy, with AUC values of 0.9443 and 0.9588, respectively.

**TABLE 1:** Performance comparison of different methods across datasets using AUC.

Datasets	LBMFF	SCPMFDD	SCPMF	MKGCN	MNGACDA	DDAGDL	RGLDR	AMFGNN
Fdataset	0.7953 ± 0.035	0.7740 ± 0.001	0.8957 ± 0.001	0.8870 ± 0.001	0.8179 ± 0.005	0.9266 ± 0.001	0.9311 ± 0.005	0.9328 ± 0.014
Cdataset	0.9069 ± 0.001	0.7937 ± 0.001	0.9117 ± 0.002	0.9109 ± 0.001	0.8406 ± 0.005	0.9256 ± 0.001	0.9359 ± 0.003	0.9443 ± 0.012
LRSSL	0.9139 ± 0.002	0.7668 ± 0.001	0.8977 ± 0.001	0.8596 ± 0.001	0.7936 ± 0.002	0.8990 ± 0.001	0.8032 ± 0.005	0.9588 ± 0.006
Avg	0.8720	0.7782	0.9017	0.8858	0.8173	0.9171	0.8901	0.9453

The results indicate that the AUC (Area Under the Curve) of the AMFGNN model outperform those of other models. This demonstrates that the AMFGNN model effectively improves the accuracy and stability of drug-disease association prediction by integrating multi-source heterogeneous information and dynamically adjusting the weights of different information sources. In addition, based on the results of Recall (Table 2) and F1-score (Table 3), AMFGNN also performs excellently in these metrics. In terms of Recall, AMFGNN effectively captures positive samples and reduces false negatives, indicating its high recall ability. A high Recall value means the model can identify more positive samples, which is especially important for drug-disease association prediction, as missing positive samples could lead to the omission of crucial information. Regarding F1-score, AMFGNN demonstrates a good balance between Precision and Recall, indicating its advantages in both accuracy and recall ability. A high F1-score means the model reduces false positives while effectively capturing more positive samples, avoiding the performance imbalance that may arise from optimizing a single metric. Overall, AMFGNN shows outstanding performance in improving prediction accuracy, stability, and comprehensiveness, further confirming its effectiveness in drug-disease association prediction, especially on complex and imbalanced datasets.

**TABLE 2:** Recall comparison of various models across multiple datasets.

Datasets	LBMFF	SCPMFDD	SCPMF	MKGCN	MNGACDA	DDAGDL	RGLDR	AMFGNN
Fdataset	0.7328	0.0255	0.4128	0.0098	0.0801	0.4560	0.8814	0.9152
Cdataset	0.7006	0.0217	0.4767	0.0644	0.1695	0.4830	0.8724	0.9443
LRSSL	0.7165	0.0151	0.3812	0.0233	0.0737	0.4328	0.6975	0.9371
Avg	0.7166	0.0208	0.4236	0.0325	0.1078	0.4573	0.8171	0.9322

**TABLE 3 F1:** -Score performance of different approaches across datasets.

Datasets	LBMFF	SCPMFDD	SCPMF	MKGCN	MNGACDA	DDAGDL	RGLDR	AMFGNN
Fdataset	0.0577	0.1174	0.4024	0.0195	0.1475	0.6007	0.8615	0.8699
Cdataset	0.2343	0.0857	0.4556	0.1210	0.2875	0.6013	0.8806	0.8890
LRSSL	0.2304	0.0249	0.3548	0.0452	0.1352	0.5538	0.7194	0.9040
Avg	0.1741	0.0760	0.4043	0.0619	0.1901	0.5853	0.8205	0.8876

**FIGURE 2:**
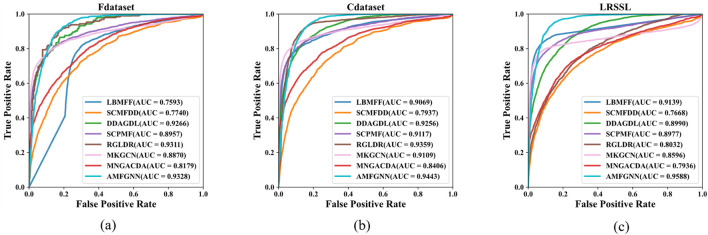
The ability of various methods to predict potential drugs for novel diseases is evaluated on public datasets using AUROC serving as the metric: **(a)** Fdataset **(b)** Cdataset **(c)** LRSSL.

As shown in Figure 2, the AUC (Area Under the Curve) values of the AMFGNN model outperform those of other models. This demonstrates that the AMFGNN model effectively improves the accuracy and stability of drug-disease association prediction by integrating multi-source heterogeneous information and dynamically adjusting the weights of different information sources.

### 3.4 Ablation studies

To thoroughly investigate the effectiveness of different components in our proposed AMFGNN model, we conduct comprehensive ablation studies. Specifically, we evaluate four variants of our model:

• Full Model: The complete AMFGNN architecture with all components, including the Kolmogorov-Arnold Networks and contrastive learning loss.
• AMFGNN w/o KAN: A variant without the Kolmogorov-Arnold Network, while maintaining the contrastive learning mechanism.
• AMFGNN w/o CL: A variant that removes the contrastive learning loss while retaining the Kolmogorov-Arnold Networks, utilizing only the main task loss function for optimization.
• AMFGNN w/o KAN & CL: The baseline variant that removes both the Kolmogorov-Arnold Network and contrastive learning loss, maintaining only the basic MLP and main task loss.

**FIGURE 3:**
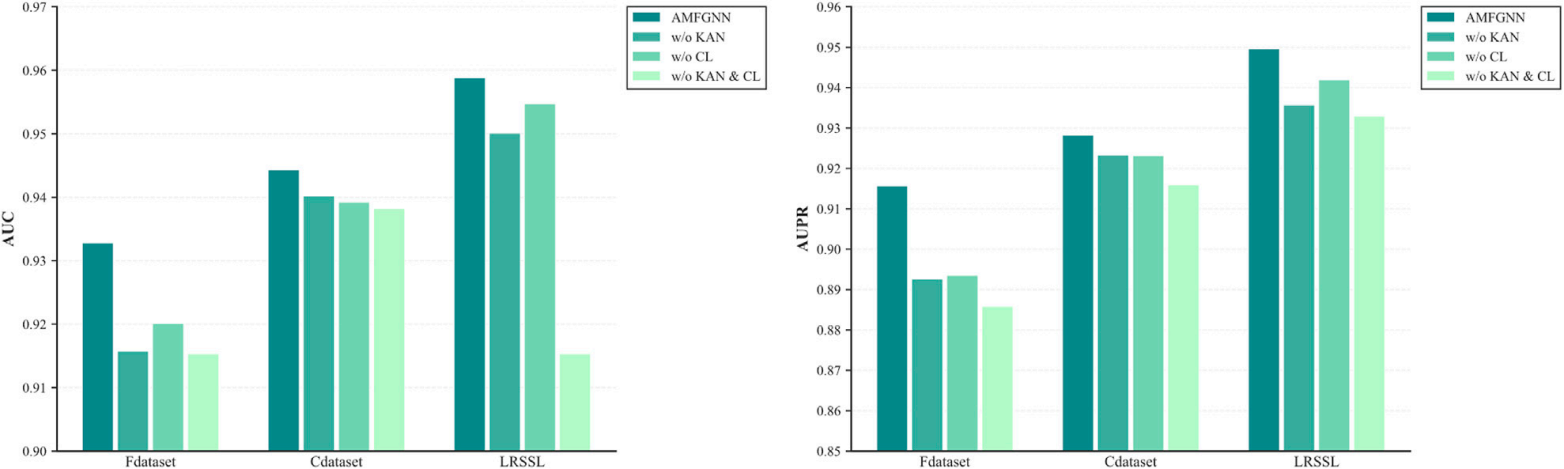
Results of ablation experiments on three datasets.

As shown in Figure 3,the experimental results demonstrate several key findings: The full AMFGNN model achieves the best performance across all metrics, validating the effectiveness of our proposed architecture. Removing the Kolmogorov-Arnold Networks (w/o KAN) leads to a performance decrease of AUC and AUPR, highlighting the importance of KAN layer in our model. The absence of contrastive learning (w/o CL) also results in a drop, indicating that the contrastive learning mechanism plays a crucial role in learning more discriminative feature representations. The baseline variant (w/o KAN & CL) shows the most significant performance degradation, confirming that both components contribute substantially to the model’s effectiveness.

These ablation studies clearly demonstrate that each component in our proposed AMFGNN makes meaningful contributions to the overall performance, and their combination leads to the optimal results.

### 3.5 Case study

To evaluate the practical application value of the model, we conducted a case study. Specifically, the model was trained on the Fdataset to predict potential drugs associated with asthma and hepatoblastoma. The predicted drugs were ranked in descending order based on their probability scores, and the top ten candidates were selected for further analysis. To assess the reliability of the model’s predictions, comprehensive validation was performed using multiple authoritative data sources, including DrugCentral, Avram et al. (2021) CTD, and ClinicalTrials databases.

Hepatoblastoma is a malignant liver tumor primarily occurring in children, typically diagnosed during infancy or early childhood (Sharma et al., 2017). Table 4 highlights five potential therapeutic agents for hepatoblastoma predicted by the AMFGNN model, four of which have been validated by authoritative databases. Furosemide, widely used in both adults and children to manage hypertension and edema caused by liver dysfunction, was identified by the AMFGNN model as a promising candidate for hepatoblastoma treatment. This prediction has been corroborated by the DrugCentral database. Furthermore, the model predicted citalopram as another potential therapeutic agent for hepatoblastoma, with this conclusion supported by both the DrugCentral and CTD databases. These findings provide new perspectives and directions for drug development targeting hepatoblastoma.

**TABLE 4 Top:** 5 candidate drugs for Hepatoblastoma predicted by AMFGNN.

Rank	DrugBank IDs	Candidate drugs	Evidences
1	DB00397	Phenylpropanolamine	DrugCentral
2	DB00313	Valproic acid	DrugCentral, CTD
3	DB00215	Citalopram	DrugCentral, CTD
4	DB00448	Lansoprazole	DrugCentral, CTD
5	DB00167	Isoleucine	Unconfirmed

Asthma is a chronic inflammatory disease influenced by both genetic and environmental factors (Toskala and Kennedy, 2015), making it a complex hereditary condition. Table 5 lists the top five potential asthma treatments predicted based on the F dataset, of which four are verified through reliable databases or clinical trials, further supporting the accuracy and practicality of the model’s predictions. AMFGNN predicts flunisolide as a potential drug for treating asthma, a conclusion supported by both DrugBank and ClinicalTrials.gov. Additionally, studies show that uncontrolled asthma is often associated with gastroesophageal reflux disease (GERD) (Harding, 2003). As a proton pump inhibitor, esomeprazole is widely used to treat GERD, and AMFGNN also predicts that esomeprazole might have therapeutic effects on asthma, a prediction that is verified by ClinicalTrials.gov.

**TABLE 5 Top:** 5 candidate drugs for asthma predicted by AMFGNN.

Rank	DrugBank IDs	Candidate drugs	Evidences
1	DB00180	Flunisolide	DB, ClinicalTrials.gov
2	DB00736	Esomeprazole	ClinicalTrials.gov
3	DB00182	Amphetamine	DrugCentral, ClinicalTrials.gov
4	DB00181	Baclofen	Unconfirmed
5	DB00695	Furosemide	ClinicalTrials.gov

Alzheimer’s Disease (AD) is a neurodegenerative disorder characterized primarily by progressive cognitive decline and memory impairmentAbubakar et al. (2022). Table 6 lists five potential drug candidates for the treatment of Alzheimer’s Disease as predicted by the AMFGNN model, four of which have already been validated by authoritative pharmaceutical databases. Memantine, an approved N-methyl-D-aspartate (NMDA) receptor antagonist, was identified by AMFGNN as an effective therapeutic agent for Alzheimer’s Disease. Additionally, Methylphenidate and Levothyroxine were also predicted as promising therapeutic candidates, with supporting evidence from clinical trials documented in the ClinicalTrials.gov database, suggesting their potential clinical application in the future. 

**TABLE 6:** TOP5 candidate drugs for Alzheimer’s Disease predicted by AMFGNN.

Rank	DrugBank IDs	Candidate drugs	Evidences
1	DB01043	Memantine	ClinicalTrials.gov, CTD, DrugCentral
2	DB00642	Methylphenidate	ClinicalTrials.gov
3	DB00636	Clofibrate	Unconfirmed
4	DB00413	Pramipexole	ClinicalTrials.gov, DrugCentral
5	DB00337	Levothyroxine	ClinicalTrials.gov

**FIGURE 4:**
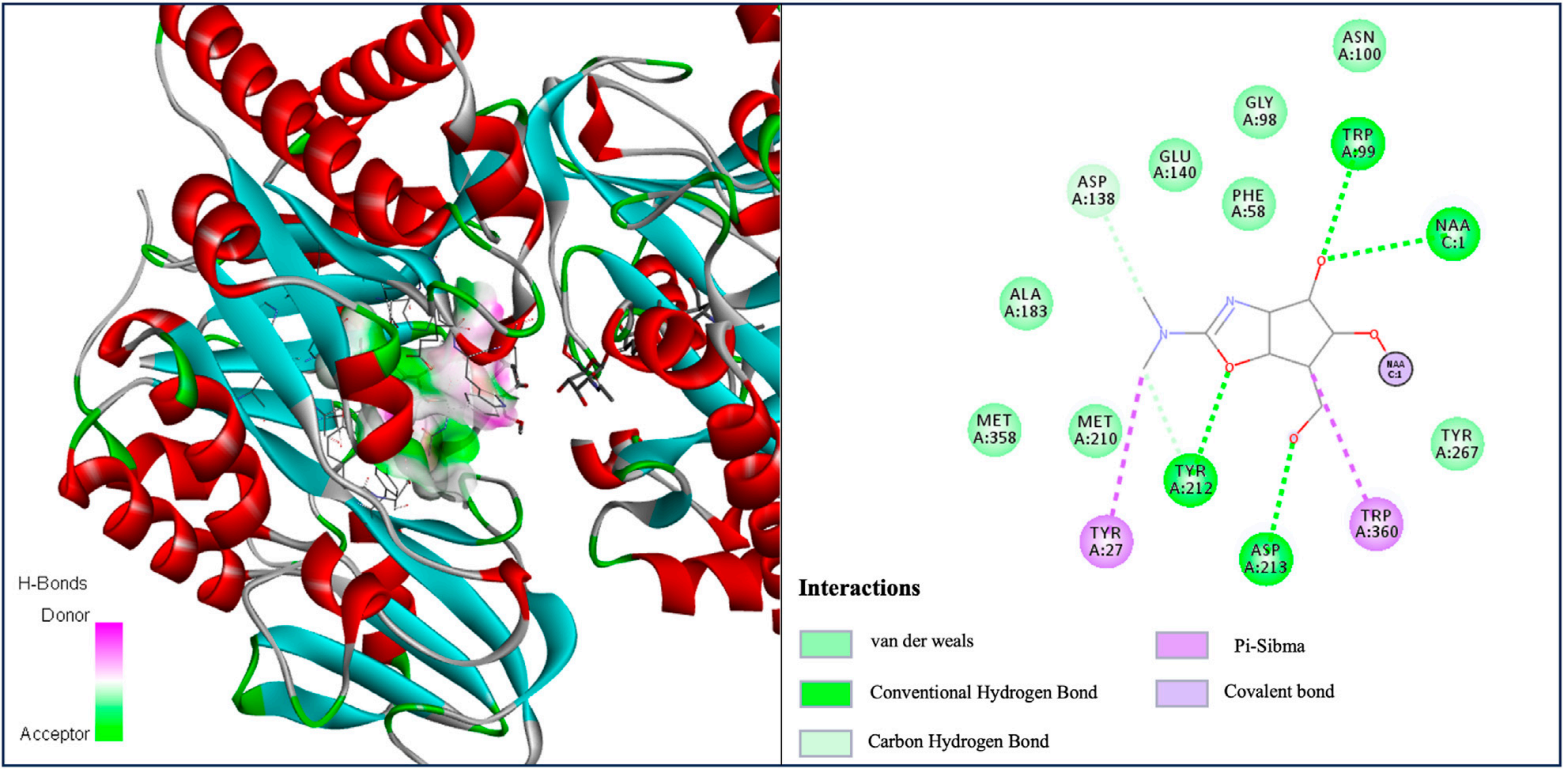
Docking and interactions of Baclofen (DrugBank ID: DB00181) with AMCase (PDB code: 3FY1).

Moreover, we selected five asthma-related target proteins and conducted molecular docking simulations to assess their binding abilities with five candidate drugs using AutoDock Vina (Trott and Olson, 2010). The interactions between the ligands and target proteins were further analyzed using Discovery Studio (DS) visualization software. Regarding the relevance of baclofen to asthma, we chose acidic mammalian chitinase (AMCase, PDB code: 3FY1) as the target protein and found that baclofen has a binding energy of −6.6 kcal/mol with AMCase (Table 7). Figure 4 shows the van der Waals interactions between baclofen and several specific amino acid residues (ALA:183, MET:385, MET:210, TYR:267, GLU:140, GLY:98, PHE:58). In addition, other types of molecular interactions are observed. For example, the oxygen atom forms conventional hydrogen bonds with residues TYR:212, ASP:213, TRP:99, and NA:1, while the nitrogen atom exhibits carbon-hydrogen bond interactions with residue ASP:138. Furthermore, π−σ interactions are observed between the small molecule and residues TYR:27 and TRP:360, and covalent bonds form between the oxygen atom and the functional group of residue NA:1.

**TABLE 7:** Molecular binding energies (kcal/mol) between the top 5 candidate drugs for asthma predicted by AMFGNN and 5 target proteins.

Drug	1KTJ	3FY1	4P0I	5BOW	7XXW
Flunisolide	−7.4	−9.6	−9.6	−7.5	−6.8
Esomeprazole	−8.0	−8.5	−6.6	−7.0	−5.9
Amphetamine	−5.7	−5.5	−4.8	−5.1	−4.4
Baclofen	−6.0	−6.6	−5.7	−6.4	−4.3
Furosemide	−6.2	−7.4	−6.2	−6.8	−5.9

## 4 Conclusion

This paper presents the Adaptive Multi-View Fusion Graph Neural Network (AMFGNN), a novel model designed for drug-disease association prediction. AMFGNN integrates multiple data sources, including drug similarity, disease similarity, and drug-disease interactions, using an adaptive multi-view feature fusion strategy. The model combines Graph Attention Networks (GAT) with contrastive learning to improve the accuracy of drug-disease predictions by effectively capturing relationships between nodes in the graph. Additionally, replacing traditional multilayer perceptron (MLP) layers with Kolmogorov-Arnold Networks (KAN) enhances the model’s flexibility, expressive capability, and overall predictive performance.

Through 10-fold cross-validation on three benchmark datasets (F dataset, C dataset, and LRSSL), AMFGNN outperforms existing models, achieving high area under the curve (AUC) scores of 0.9328, 0.9443, and 0.9588, respectively. These results demonstrate that AMFGNN significantly improves drug-disease prediction accuracy, making it a valuable tool in drug repositioning and personalized medicine.

## Data Availability

The original contributions presented in the study are included in the article/supplementary material, further inquiries can be directed to the corresponding authors.
